# Adolescent pregnancy, nutrition, and health outcomes in low‐ and middle‐income countries: what we know and what we don't know

**DOI:** 10.1111/1471-0528.13782

**Published:** 2015-12-02

**Authors:** W Johnson, SE Moore

**Affiliations:** ^1^ Elsie Widdowson LaboratoryMedical Research Council (MRC) Human Nutrition ResearchCambridgeUK

Adolescence [defined by the World Health Organization (WHO) as the time period between the ages of 10 and 19 years] is a critical period in human physical and psychosocial development when an individual progresses from an immature state to a mature state capable of reproduction. Pregnancies in this stage of life account for 23% of the burden of disease arising from pregnancy and childbirth, despite only representing 11% of all births worldwide.^1^ They incur increased risks for a number of adverse growth and developmental outcomes, in both the offspring (e.g. small for gestational age, SGA)^2^, ^3^ and the mother (e.g. early cessation of linear growth),^4^, ^5^ that are known to impact adversely on long‐term morbidity and mortality risk.^3^, ^6^

Ninety‐five percent of the 16 million adolescent pregnancies that occur each year are in low‐ and middle‐income countries (LMICs),^1^ and this is where the burden of SGA and stunting is concentrated.^7^ *BJOG* published an issue last year on the WHO Multicountry Survey of Maternal and Newborn Health, in which the authors of one paper reported higher rates of various pregnancy and childbirth outcomes (including low birthweight) in adolescents aged 10–19 years, compared with young adults aged 20–24 years, and concluded that ‘interventions are crucial to reduce adverse pregnancy outcomes among adolescent women in LMICs’.^2^ Given the close links of nutrition with growth and development and the high burden of undernutrition in many of these settings,^8^ we questioned what evidence exists to design a tailored nutritional intervention. This commentary reviews what we know and don't know about the nutritional determinants of the adverse growth and development‐related health outcomes of adolescent pregnancy, drawing on evidence firstly from observation studies and secondly from intervention studies. We end by discussing the need for more robustly designed observational studies to understand the nutritional epidemiology of adolescent pregnancy, and provide a stronger evidence base against which future nutritional interventions can be developed.

## Evidence from observation studies

Physical size (e.g. weight and height) is an indicator of nutritional status, and the odds of SGA are increased in adult women with pre‐pregnancy height [odds ratio (OR) 1.9] or weight (OR 2.5) in the lowest, compared with the highest, quartile.^9^ The well‐known increased risk of adverse birth outcomes in pregnant adolescents, compared with pregnant young adults,^2^, ^3^ coincides with the fact that adolescents are smaller because they are still growing: between menarche and the cessation of linear growth approximately an additional 7 cm in height is gained, on average.^10^ It is hypothesised that there is competition for nutrients between the still growing adolescent mother and her rapidly developing fetus, also known as ‘nutrient partitioning’, which may result in the growth and development of the mother and/or fetus being compromised. An alternative explanation, which may work in tandem with nutrient partitioning, is that optimal fetal development is being traded‐off as a result of gynaecological immaturity (in girls who are still growing and developing) to allow safe delivery. This rationale is supported by the evidence that the risks of SGA are greatest in girls who are the most gynaecologically immature.^11^

Studies in Bangladesh and Mexico have suggested that adolescent girls (aged 12–19 years and 13–17 years, respectively) stop growing in response to pregnancy: the change in height (from the first trimester to 6 months postpartum in the study from Bangladesh, and from <20 weeks of gestation to 1 month postpartum in the study from Mexico) was approximately zero in pregnant adolescent girls, but was positive and significant in non‐pregnant adolescent girls matched on age and menarcheal age.^4^, ^5^ Our group have shown that adolescence offers a window of opportunity for catch‐up growth in response to early life stunting.^12^ Given our knowledge of the consequences of short stature in adulthood for a wide array of human capital and health outcomes, including increased all‐cause mortality,^6^ there is a clear need to understand the modifiable nutritional factors that adversely affect linear growth in pregnant adolescent girls. Between‐group differences in the study from Bangladesh remained significant after adjustment for dietary intake (assessed by a food‐frequency questionnaire), but that does not rule out inadequate nutritional supply as the limiting factor compromising adolescent growth, as this simple adjustment doesn't account for the additional requirements needed for pregnancy and lactation.

The two prominent adolescent pregnancy nutrient‐partitioning studies that have investigated offspring outcomes were conducted in high‐income countries (HICs).^13^, ^14^ Both studies compared differences in nutritional status and adverse birth outcomes between growing adolescents and a non‐growing referent group, in whom the nutritional costs of growth and thus any competition for nutrients were argued to be diminished. In the USA, the Camden Study found lower birthweights and higher rates of preterm delivery in growing (defined as a change in knee height of >1 mm over 6 months, from the second trimester to 6 weeks postpartum) compared with non‐growing females aged 12–18 years; effect sizes were greatest in younger girls, those of multiparous gravida, and those with the lowest energy intakes.^14^ Conversely, the About Teenage Eating (ATE) study in the UK found that growing (defined as a change in knee height of >2 mm over 90 days, from 13 to 29 weeks of gestation), mainly nulliparous adolescents aged 14–18 years actually had more large‐for‐gestational age babies compared with their non‐growing peers.^13^ In both studies, nutrient intakes generally exceeded recommended values, and there were no differences in macronutrient intakes (ATE study only) or energy intake between the growing and non‐growing adolescent groups. Results for micronutrients were equivocal, but did suggest that (compared with non‐growing adolescents) growing adolescents had poorer nutritional status in the Camden Study (e.g. lower maternal and cord ferritin and folate), but better nutritional status in the ATE study (e.g. higher maternal folate and intakes of calcium, magnesium, phosphorus, iron, and riboflavin), which might go some way towards explaining the observed differences in birth outcomes.

A recent publication of the Consortium of Health Orientated Research in Transitioning Societies group found that adolescent pregnancy (ages ≤ 19 years), compared to young‐adulthood pregnancy (ages 20–24 years), was associated with increased risk of stunting at age 2 years (OR 1.46) and higher adulthood fasting glucose concentrations in the offspring.^15^ Besides this publication, little is known about the long‐term health consequences of adolescent pregnancy in LMICs, and what early‐life nutritional factors might offset any risk.

## Evidence from intervention studies

Six studies were identified that had supplemented the diet of pregnant adolescents and measured growth and development‐related outcomes. Two of these studies involved calcium supplementation,^16^, ^17^ one involved supplementation with calcium plus vitamin D,^18^ two involved supplementation with zinc,^19^, ^20^ and one included four intervention arms in females of childbearing age (i.e. including adults as well as adolescents), with supplementation of: (1) folic acid; (2) folic acid and iron; (3) folic acid, iron, and zinc; and (4) multiple micronutrients.^21^ Despite the differences in study populations (Brazil, Chile, Nepal, and USA), sample sizes (ranging from 30 to 705 in an intervention arm), maternal ages at baseline (e.g. ages 15–17 years or ages 13.5–19.6 years), and timing of interventions (e.g. initiation at mean gestations of 11 or 26 weeks), five out of the six studies provided some evidence of a positive effect of intervention on estimated fetal weight and/or birthweight. The study of Christian et al.^21^, for example, found that multiple prenatal micronutrient supplementation in females of childbearing age increased birthweight by 64 g (95% confidence interval: 12–115 g), although the effect size in adolescent gravida alone was not reported. Evidence of an effect on other offspring outcomes (e.g. preterm delivery and bone mineral content) included in these six studies is less conclusive, as is evidence of an effect on maternal and longer‐term health outcomes.^22^

## We need a more robust evidence base

As is clear, the literature on the nutritionally mediated pathways underpinning the links between young maternal age and poor intergenerational and long‐term health is sparse. Existing publications have studied different age ranges, without equal distribution of adolescents across those age ranges, which combined with other between‐study differences (e.g. parity) has contributed to largely equivocal findings. Evidence of nutrient partitioning is mostly limited to a few studies in HICs, where the nutritional requirements of adolescence and pregnancy are likely to be met. Two studies in LMICs suggest that adolescent girls stop growing in response to pregnancy,^4^, ^5^ but investigation at the mechanistic level using biomarkers and body‐imaging technologies is missing from the literature. Intervention studies have shown some promising results of increasing birthweight, with effect sizes not dissimilar to those reported in prenatal supplementation studies in adults.^23^ What we don't know is whether or not the SGA phenotype, and thus health risks over the course of life, are the same regardless of maternal age. This would require detailed phenotyping, including the assessment of neonatal body composition and long‐term follow‐up.

To understand which (and how and when) components of nutrition increase risk for adverse outcomes in the offspring of adolescent compared to young‐adulthood pregnancies, a prospective observational study with a young‐adult reference group is required; recruitment of equal numbers of females in each yearly age band between 13 and 26 years would provide an adolescent group and a young‐adult group, and allow in‐depth investigation of difference within these groups. Furthermore, to understand which components of nutrition increase risk for adverse outcomes in the adolescents themselves (and how and when), a non‐pregnant reference group matched on key variables (e.g. age, menarcheal age, and parity) is also required. The females and their offspring would ideally be followed‐up for long‐term health assessment and subsequent pregnancies. Such a study would be best suited to LMICs where rates of adolescent pregnancy and their adverse sequelae are high. Preferably, a multicentre programme of research across contrasting environments (in the same country or in different countries) would be conducted in order to understand how the nutritional epidemiology of adolescent pregnancy might depend on context (e.g. the disease profile of the population being studied). This will enable any subsequent nutritional intervention to be appropriately tailored. Careful consideration of the context of adolescent pregnancy within each setting would also be necessary to understand the role of socio‐economic position in influencing both the probability of an adolescent becoming pregnant and (via nutrition and other factors) the risk of adverse outcomes.^24^ For these reasons, a nutritional intervention may best be implemented as part of a multifaceted programme, including nutritional and sexual health education.

Although the WHO recommend pregnancy prevention as the primary solution for poor reproductive outcomes of adolescent pregnancy, societal and cultural practices in many LMICs are a barrier to changes in practice.^25^ Nutrition is widely recognised as a key target for improving adolescent health, and the health of their offspring,^26^, ^27^ yet the evidence base for developing an intervention targeting this large and accessible population group who are preparing for pregnancy is limited. We call for a reinvigorated and coordinated effort across multiple LMIC settings to understand the nutritional epidemiology of adolescent pregnancy, and its sequelae, where the disease burden is greatest.

### Disclosure of interests

None declared. Completed disclosure of interests form available to view online as supporting information.

### Contribution to authorship

WJ and SEM conceptualized the commentary, drafted and revised the article critically for important intellectual content, approved the version to be published, and agree to be accountable for all aspects of the work.

### Details of ethics approval

Not applicable.

### Funding

WJ and SEM are funded by the Medical Research Council (UK) programme: MC_UP_1005/1.

## References

[1] World Health Organisation . Adolescent pregnancy. Fact sheet No. 364 2014. [www.who.int/mediacentre/factsheets/fs364/en/]. Accessed 4 June 2015.

[2] Ganchimeg T , Ota E , Morisaki N , Laopaiboon M , Lumbiganon P , Zhang J , et al. Pregnancy and childbirth outcomes among adolescent mothers: a World Health Organization multicountry study. BJOG 2014;121(Suppl 1):40–8. 24641534 10.1111/1471-0528.12630

[3] Kozuki N , Lee AC , Silveira MF , Sania A , Vogel JP , Adair L , et al. The associations of parity and maternal age with small‐for‐gestational‐age, preterm, and neonatal and infant mortality: a meta‐analysis. BMC Public Health 2013;13(Suppl 3):S2. 24564800 10.1186/1471-2458-13-S3-S2 PMC3847520

[4] Casanueva E , Rosello‐Soberon ME , De‐Regil LM , Arguelles Mdel C , Cespedes MI . Adolescents with adequate birth weight newborns diminish energy expenditure and cease growth. J Nutr 2006;136:2498–501. 16988116 10.1093/jn/136.10.2498

[5] Rah JH , Christian P , Shamim AA , Arju UT , Labrique AB , Rashid M . Pregnancy and lactation hinder growth and nutritional status of adolescent girls in rural Bangladesh. J Nutr 2008;138:1505–11. 18641198 10.1093/jn/138.8.1505

[6] Emerging Risk Factors Collaboration . Adult height and the risk of cause‐specific death and vascular morbidity in 1 million people: individual participant meta‐analysis. Int J Epidemiol 2012;41:1419–33. 22825588 10.1093/ije/dys086 PMC3465767

[7] Lee AC , Katz J , Blencowe H , Cousens S , Kozuki N , Vogel JP , et al. National and regional estimates of term and preterm babies born small for gestational age in 138 low‐income and middle‐income countries in 2010. Lancet Glob Health 2013;1:e26–36. 25103583 10.1016/S2214-109X(13)70006-8 PMC4221634

[8] Black RE , Victora CG , Walker SP , Bhutta ZA , Christian P , de Onis M , et al. Maternal and child undernutrition and overweight in low‐income and middle‐income countries. Lancet 2013;382:427–51. 23746772 10.1016/S0140-6736(13)60937-X

[9] World Health Organisation . Maternal anthropometry and pregnancy outcomes. A WHO Collaborative Study. Bull World Health Organ 1995;73(Suppl):1–98. PMC2486648 8529277

[10] Roche AF , Davila GH . Late adolescent growth in stature. Pediatrics 1972;50:874–80. 4673900

[11] Kaplanoglu M , Bulbul M , Konca C , Kaplanoglu D , Tabak MS , Ata B . Gynecologic age is an important risk factor for obstetric and perinatal outcomes in adolescent pregnancies. Women Birth 2015; In Press. 10.1016/j.wombi.2015.07.002 26205092

[12] Prentice AM , Ward KA , Goldberg GR , Jarjou LM , Moore SE , Fulford AJ , et al. Critical windows for nutritional interventions against stunting. Am J Clin Nutr 2013;97:911–8. 23553163 10.3945/ajcn.112.052332 PMC3628381

[13] Jones RL , Cederberg HM , Wheeler SJ , Poston L , Hutchinson CJ , Seed PT , et al. Relationship between maternal growth, infant birthweight and nutrient partitioning in teenage pregnancies. BJOG 2010;117:200–11. 19832832 10.1111/j.1471-0528.2009.02371.x

[14] Scholl TO , Hediger ML , Schall JI . Maternal growth and fetal growth: pregnancy course and outcome in the Camden Study. Ann N Y Acad Sci 1997;817:292–301. 9239197 10.1111/j.1749-6632.1997.tb48215.x

[15] Fall CH , Sachdev HS , Osmond C , Restrepo‐Mendez MC , Victora C , Martorell R , et al. Association between maternal age at childbirth and child and adult outcomes in the offspring: a prospective study in five low‐income and middle‐income countries (COHORTS collaboration). Lancet Glob Health 2015;3:e366–77. 25999096 10.1016/S2214-109X(15)00038-8 PMC4547329

[16] Chan GM , McElligott K , McNaught T , Gill G . Effects of dietary calcium intervention on adolescent mothers and newborns: a randomized controlled trial. Obstet Gynecol 2006;108(3 Pt 1):565–71. 16946216 10.1097/01.AOG.0000231721.42823.9e

[17] Villar J , Repke JT . Calcium supplementation during pregnancy may reduce preterm delivery in high‐risk populations. Am J Obstet Gynecol 1990;163(4 Pt 1):1124–31. 2220915 10.1016/0002-9378(90)90669-x

[18] Diogenes ME , Bezerra FF , Rezende EP , Donangelo CM . Calcium plus vitamin D supplementation during the third trimester of pregnancy in adolescents accustomed to low calcium diets does not affect infant bone mass at early lactation in a randomized controlled trial. J Nutr 2015;145:1515–23. 26019245 10.3945/jn.114.208140

[19] Cherry FF , Sandstead HH , Rojas P , Johnson LK , Batson HK , Wang XB . Adolescent pregnancy: associations among body weight, zinc nutriture, and pregnancy outcome. Am J Clin Nutr 1989;50:945–54. 2816802 10.1093/ajcn/50.5.945

[20] Hunt IF , Murphy NJ , Cleaver AE , Faraji B , Swendseid ME , Browdy BL , et al. Zinc supplementation during pregnancy in low‐income teenagers of Mexican descent: effects on selected blood constituents and on progress and outcome of pregnancy. Am J Clin Nutr 1985;42:815–28. 4061343 10.1093/ajcn/42.5.815

[21] Christian P , Khatry SK , Katz J , Pradhan EK , LeClerq SC , Shrestha SR , et al. Effects of alternative maternal micronutrient supplements on low birth weight in rural Nepal: double blind randomised community trial. BMJ 2003;326:571. 12637400 10.1136/bmj.326.7389.571 PMC151518

[22] Diogenes ME , Bezerra FF , Rezende EP , Taveira MF , Pinhal I , Donangelo CM . Effect of calcium plus vitamin D supplementation during pregnancy in Brazilian adolescent mothers: a randomized, placebo‐controlled trial. Am J Clin Nutr 2013;98:82–91. 23719547 10.3945/ajcn.112.056275

[23] Fall CH , Fisher DJ , Osmond C , Margetts BM , Maternal Micronutrient Supplementation Study Group . Multiple micronutrient supplementation during pregnancy in low‐income countries: a meta‐analysis of effects on birth size and length of gestation. Food Nutr Bull 2009;30(4 Suppl):S533–46. 20120795 10.1177/15648265090304S408 PMC3541502

[24] Restrepo‐Mendez MC , Lawlor DA , Horta BL , Matijasevich A , Santos IS , Menezes AM , et al. The association of maternal age with birthweight and gestational age: a cross‐cohort comparison. Paediatr Perinat Epidemiol 2015;29:31–40. 25405673 10.1111/ppe.12162 PMC4296235

[25] WHO . WHO Guidelines on Preventing Early Pregnancy and Poor Reproductive Outcomes Among Adolescents in Developing Countries. Geneva, Switzerland: WHO, 2011. 10.1016/j.jadohealth.2013.03.002 23608717

[26] Blum RW , Bastos FI , Kabiru CW , Le LC . Adolescent health in the 21st century. Lancet 2012;379:1567–8. 22538177 10.1016/S0140-6736(12)60407-3

[27] Patton GC , Ross DA , Santelli JS , Sawyer SM , Viner RM , Kleinert S . Next steps for adolescent health: a Lancet Commission. Lancet 2014;383:385–6. 24485572 10.1016/S0140-6736(14)60039-8

